# 2,6-Bis(2,4-dimethyl­benzyl­idene)cyclo­hexa­none

**DOI:** 10.1107/S1600536810018684

**Published:** 2010-05-22

**Authors:** Katarzyna A. Solanko, Andrew D. Bond

**Affiliations:** a University of Southern Denmark, Department of Physics and Chemistry, Campusvej 55, 5230 Odense, Denmark

## Abstract

In the crystal structure of the title compound, C_24_H_6_O, the mol­ecule exhibits point symmetry *m* but the mirror plane is not utilized as part of the space-group symmetry. The structure contains face-to-face inter­actions between the 2,4-dimethyl­benzyl­idene substituents in which the methyl groups lie directly above the centroids of adjacent benzene rings.

## Related literature

For related structures, see: Guo *et al.* (2008 ▶); Jia *et al.* (1989 ▶); Liu (2009 ▶); Ompraba *et al.* (2003 ▶); Shi *et al.* (2008 ▶); Zhang *et al.* (2005 ▶); Zhou (2007 ▶). For quanti­fication of the mol­ecular point symmetry, see: Pilati & Forni (1998 ▶, 2000 ▶).
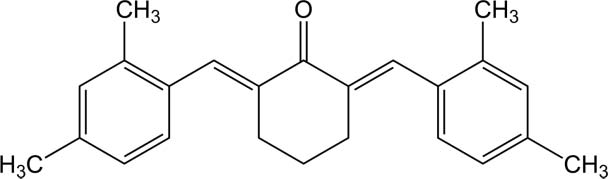


## Experimental

### 

#### Crystal data

C_24_H_26_O
*M* *_r_* = 330.45
Monoclinic, 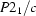

*a* = 6.9784 (4) Å
*b* = 19.2540 (12) Å
*c* = 14.2829 (10) Å
β = 102.179 (3)°
*V* = 1875.9 (2) Å^3^
*Z* = 4
Mo *K*α radiation
μ = 0.07 mm^−1^
*T* = 120 K
0.60 × 0.20 × 0.20 mm

#### Data collection

Bruker–Nonius X8 APEXII CCD diffractometer
Absorption correction: multi-scan (*SADABS*; Bruker, 2003 ▶) *T*_min_ = 0.895, *T*_max_ = 0.986
32106 measured reflections
3565 independent reflections
2399 reflections with *I* > 2σ(*I*)
*R*_int_ = 0.042

#### Refinement

*R*[*F* ^2^ > 2σ(*F* ^2^)] = 0.040
*wR*(*F* ^2^) = 0.113
*S* = 1.08
3565 reflections
230 parameters
H-atom parameters constrained
Δρ_max_ = 0.21 e Å^−3^
Δρ_min_ = −0.22 e Å^−3^

### 

Data collection: *APEX2* (Bruker, 2004 ▶); cell refinement: *SAINT* (Bruker, 2003 ▶); data reduction: *SAINT*; program(s) used to solve structure: *SHELXTL* (Sheldrick, 2008 ▶); program(s) used to refine structure: *SHELXTL*; molecular graphics: *SHELXTL*; software used to prepare material for publication: *SHELXTL*.

## Supplementary Material

Crystal structure: contains datablocks global, I. DOI: 10.1107/S1600536810018684/jh2159sup1.cif

Structure factors: contains datablocks I. DOI: 10.1107/S1600536810018684/jh2159Isup2.hkl

Additional supplementary materials:  crystallographic information; 3D view; checkCIF report

## Figures and Tables

**Table 1:** C—H⋯π interactions (Å, °) *Cg*1 and *Cg*2 are the centroids of the C21–C26 and C11–C16 rings, respectively.

*D*—H⋯*A*	*D*—H	H⋯*A*	*D*⋯*A*	*D*—H⋯*A*
C17—H17*B*⋯*Cg*1^i^	0.98	3.00	3.532 (1)	154
C17—H17*C*⋯*Cg*1^ii^	0.98	2.62	3.469 (1)	111
C27—H27*B*⋯*Cg*2^iii^	0.98	2.64	3.486 (1)	145
C27—H27*C*⋯*Cg*2^iv^	0.98	2.80	3.510 (1)	130

Symmetry codes: (i) 
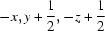
; (ii) 
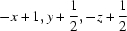
; (iii) 
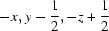
; (iv) 
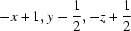
.

**Fig. 1.**
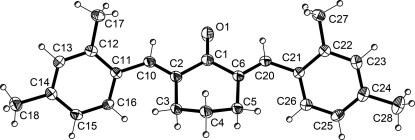
Molecular unit showing displacement ellipsoids at 50% probability. H atoms are shown as spheres of arbitrary radius.

**Fig. 2.**
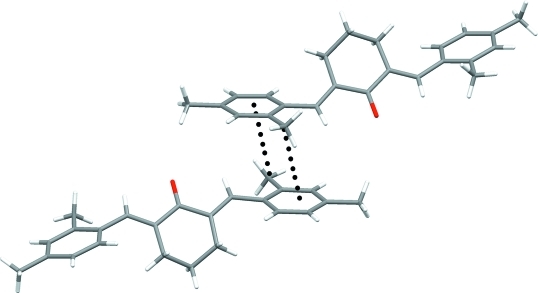
Face-to-face interactions between the 2,4-dimethylbenzylidene substituents, with C(methyl)···centroid interactions highlighted.

## supplementary crystallographic information

### Comment

We were interested in the crystal structure of 2,6-bis(2,4-dichlorobenzylidene)cyclohexanone (Guo *et al.*, 2008) because we have found that it exhibits a relatively large change in structure on cooling from room temperature to 100 K (Solanko & Bond, unpublished results). We synthesised the analogous tetra-methyl-substituted compound to examine whether it might form a similar structure and display similar behaviour. It does not.

We note that in the publication of Guo *et al.* (2008), the chloro compound is stated to be synthesised by reaction of 2,4-dichlorobenzophenone with cyclohexanone. It seems likely that this should be 2,4-dichlorobenzaldehyde with cyclohexanone, as described here in the Experimental section.

The molecular point symmetry *m* referred to in the Abstract was quantified using the program *SYMMOL* (Pilati & Forni, 1998, 2000): the rms deviation of the molecule from its *m* symmetrised counterpart is 0.055 Å.

### Experimental

2,4-Dimethylbenzaldehyde (2.8 ml, 0.02 mol), cyclohexanone (1.0 ml, 0.01 mol) and 30% NaOH(aq) (1 ml) were stirred in ethanol (3 ml) at room temperature for 6 h. The yellow product was filtered and washed using EtOH (3 × 2 ml). Crystals were obtained by slow evaporation from acetone under ambient conditions.

### Refinement

H atoms bound to C atoms were positioned geometrically and allowed to ride during subsequent refinement with C—H = 0.95–0.98 Å, and with *U*_iso_(H) = 1.2 or 1.5 *U*_eq_(C). Methyl groups were allowed to rotate about their local threefold axes.

### Crystal data

**Table:**

C_24_H_26_O	*F*(000) = 712
*M**_r_* = 330.45	*D*_x_ = 1.170 Mg m^−^^3^
Monoclinic, *P*2_1_/*c*	Mo *K*α radiation, λ = 0.71073 Å
Hall symbol: -P 2ybc	Cell parameters from 5996 reflections
*a* = 6.9784 (4) Å	θ = 2.6–24.5°
*b* = 19.2540 (12) Å	µ = 0.07 mm^−^^1^
*c* = 14.2829 (10) Å	*T* = 120 K
β = 102.179 (3)°	Needle, yellow
*V* = 1875.9 (2) Å^3^	0.60 × 0.20 × 0.20 mm
*Z* = 4	

### Data collection

**Table:**

Bruker–Nonius X8 APEXII CCD diffractometer	3565 independent reflections
Radiation source: fine-focus sealed tube	2399 reflections with *I* > 2σ(*I*)
graphite	*R*_int_ = 0.042
ω and φ scans	θ_max_ = 25.8°, θ_min_ = 3.6°
Absorption correction: multi-scan (*SADABS*; Bruker, 2003)	*h* = −8→8
*T*_min_ = 0.895, *T*_max_ = 0.986	*k* = −19→23
32106 measured reflections	*l* = −17→16

### Refinement

**Table:**

Refinement on *F*^2^	Primary atom site location: structure-invariant direct methods
Least-squares matrix: full	Secondary atom site location: difference Fourier map
*R*[*F*^2^ > 2σ(*F*^2^)] = 0.040	Hydrogen site location: inferred from neighbouring sites
*wR*(*F*^2^) = 0.113	H-atom parameters constrained
*S* = 1.08	*w* = 1/[σ^2^(*F*_o_^2^) + (0.0604*P*)^2^ + 0.133*P*] where *P* = (*F*_o_^2^ + 2*F*_c_^2^)/3
3565 reflections	(Δ/σ)_max_ < 0.001
230 parameters	Δρ_max_ = 0.21 e Å^−^^3^
0 restraints	Δρ_min_ = −0.22 e Å^−^^3^

### Special details

**Table:**

Geometry. All esds (except the esd in the dihedral angle between two l.s. planes) are estimated using the full covariance matrix. The cell esds are taken into account individually in the estimation of esds in distances, angles and torsion angles; correlations between esds in cell parameters are only used when they are defined by crystal symmetry. An approximate (isotropic) treatment of cell esds is used for estimating esds involving l.s. planes.
Refinement. Refinement of *F*^2^ against ALL reflections. The weighted *R*-factor wR and goodness of fit *S* are based on *F*^2^, conventional *R*-factors *R* are based on *F*, with *F* set to zero for negative *F*^2^. The threshold expression of *F*^2^ > σ(*F*^2^) is used only for calculating *R*-factors(gt) etc. and is not relevant to the choice of reflections for refinement. *R*-factors based on *F*^2^ are statistically about twice as large as those based on *F*, and *R*- factors based on ALL data will be even larger.

### Fractional atomic coordinates and isotropic or equivalent isotropic displacement parameters (Å^2^)

**Table:**

	*x*	*y*	*z*	*U*_iso_*/*U*_eq_	
O1	0.0853 (2)	0.39396 (5)	0.26683 (9)	0.0545 (4)	
C1	0.1796 (2)	0.39689 (7)	0.20350 (11)	0.0297 (4)	
C2	0.2281 (2)	0.46589 (7)	0.16524 (10)	0.0243 (3)	
C3	0.3539 (2)	0.46722 (7)	0.09156 (10)	0.0250 (4)	
H3A	0.4324	0.5105	0.0989	0.030*	
H3B	0.2687	0.4673	0.0267	0.030*	
C4	0.4907 (2)	0.40496 (7)	0.10168 (11)	0.0274 (4)	
H4A	0.5727	0.4074	0.0531	0.033*	
H4B	0.5785	0.4051	0.1659	0.033*	
C5	0.3697 (2)	0.33903 (7)	0.08813 (10)	0.0246 (4)	
H5A	0.2836	0.3391	0.0234	0.030*	
H5B	0.4583	0.2985	0.0924	0.030*	
C6	0.24585 (19)	0.33209 (7)	0.16204 (10)	0.0226 (3)	
C10	0.1637 (2)	0.52272 (7)	0.20284 (10)	0.0243 (4)	
H10A	0.0927	0.5149	0.2518	0.029*	
C11	0.18876 (19)	0.59566 (7)	0.17793 (10)	0.0205 (3)	
C12	0.23238 (18)	0.64565 (7)	0.25081 (10)	0.0207 (3)	
C13	0.25309 (18)	0.71416 (7)	0.22573 (10)	0.0215 (3)	
H13A	0.2840	0.7477	0.2754	0.026*	
C14	0.23096 (18)	0.73622 (7)	0.13169 (10)	0.0227 (3)	
C15	0.18354 (19)	0.68668 (7)	0.05996 (10)	0.0225 (3)	
H15A	0.1646	0.7003	−0.0053	0.027*	
C16	0.16363 (19)	0.61763 (7)	0.08281 (10)	0.0217 (3)	
H16A	0.1322	0.5844	0.0328	0.026*	
C17	0.2599 (2)	0.62591 (8)	0.35419 (10)	0.0271 (4)	
H17A	0.3140	0.6655	0.3944	0.041*	
H17B	0.3505	0.5865	0.3677	0.041*	
H17C	0.1332	0.6128	0.3682	0.041*	
C18	0.2627 (2)	0.81108 (8)	0.11040 (11)	0.0330 (4)	
H18A	0.2040	0.8406	0.1527	0.049*	
H18B	0.2012	0.8211	0.0435	0.049*	
H18C	0.4036	0.8205	0.1210	0.049*	
C20	0.19241 (19)	0.27140 (7)	0.19475 (10)	0.0225 (3)	
H20A	0.1265	0.2743	0.2464	0.027*	
C21	0.22247 (18)	0.20107 (7)	0.16113 (9)	0.0198 (3)	
C22	0.25798 (18)	0.14544 (7)	0.22633 (10)	0.0199 (3)	
C23	0.27183 (19)	0.07907 (7)	0.19172 (10)	0.0247 (4)	
H23A	0.2970	0.0417	0.2361	0.030*	
C24	0.2504 (2)	0.06452 (8)	0.09459 (11)	0.0278 (4)	
C25	0.2156 (2)	0.11958 (8)	0.03073 (11)	0.0268 (4)	
H25A	0.2004	0.1113	−0.0360	0.032*	
C26	0.20303 (19)	0.18648 (8)	0.06376 (10)	0.0239 (4)	
H26A	0.1805	0.2236	0.0190	0.029*	
C27	0.28382 (19)	0.15735 (8)	0.33191 (9)	0.0255 (4)	
H27A	0.3023	0.1127	0.3655	0.038*	
H27B	0.1671	0.1804	0.3450	0.038*	
H27C	0.3990	0.1868	0.3543	0.038*	
C28	0.2652 (3)	−0.00880 (8)	0.06076 (13)	0.0441 (5)	
H28A	0.1693	−0.0379	0.0835	0.066*	
H28B	0.3975	−0.0266	0.0861	0.066*	
H28C	0.2384	−0.0097	−0.0094	0.066*	

### Atomic displacement parameters (Å^2^)

**Table:**

	*U*^11^	*U*^22^	*U*^33^	*U*^12^	*U*^13^	*U*^23^
O1	0.0908 (10)	0.0269 (7)	0.0671 (9)	−0.0005 (6)	0.0645 (8)	0.0014 (6)
C1	0.0368 (9)	0.0268 (10)	0.0313 (9)	−0.0015 (7)	0.0202 (7)	0.0008 (7)
C2	0.0257 (8)	0.0240 (9)	0.0256 (8)	0.0008 (6)	0.0105 (6)	0.0022 (7)
C3	0.0293 (8)	0.0223 (9)	0.0276 (8)	−0.0005 (6)	0.0154 (6)	0.0029 (6)
C4	0.0286 (8)	0.0266 (9)	0.0316 (9)	0.0009 (7)	0.0170 (7)	0.0030 (7)
C5	0.0292 (8)	0.0232 (9)	0.0242 (8)	0.0044 (6)	0.0117 (6)	0.0032 (6)
C6	0.0247 (8)	0.0225 (9)	0.0220 (8)	0.0002 (6)	0.0080 (6)	0.0012 (6)
C10	0.0251 (8)	0.0258 (9)	0.0255 (8)	0.0013 (6)	0.0130 (6)	0.0022 (7)
C11	0.0153 (7)	0.0233 (9)	0.0251 (9)	0.0033 (6)	0.0095 (6)	0.0033 (7)
C12	0.0127 (7)	0.0269 (9)	0.0231 (8)	0.0042 (6)	0.0055 (6)	0.0022 (7)
C13	0.0171 (7)	0.0232 (9)	0.0237 (8)	0.0020 (6)	0.0034 (6)	−0.0026 (6)
C14	0.0171 (7)	0.0229 (8)	0.0283 (9)	0.0038 (6)	0.0056 (6)	0.0047 (7)
C15	0.0203 (7)	0.0262 (9)	0.0217 (8)	0.0058 (6)	0.0062 (6)	0.0059 (7)
C16	0.0195 (7)	0.0249 (9)	0.0218 (8)	0.0027 (6)	0.0070 (6)	−0.0024 (6)
C17	0.0236 (8)	0.0331 (9)	0.0252 (8)	0.0007 (7)	0.0065 (6)	0.0032 (7)
C18	0.0369 (9)	0.0268 (9)	0.0350 (10)	0.0000 (7)	0.0071 (7)	0.0045 (7)
C20	0.0233 (7)	0.0252 (9)	0.0201 (8)	−0.0003 (6)	0.0071 (6)	0.0001 (6)
C21	0.0162 (7)	0.0220 (8)	0.0218 (8)	−0.0019 (6)	0.0051 (6)	−0.0016 (6)
C22	0.0115 (7)	0.0241 (9)	0.0245 (8)	−0.0026 (6)	0.0044 (6)	0.0000 (7)
C23	0.0170 (7)	0.0222 (9)	0.0353 (10)	−0.0009 (6)	0.0060 (6)	0.0031 (7)
C24	0.0190 (7)	0.0254 (9)	0.0401 (10)	−0.0016 (6)	0.0089 (7)	−0.0071 (8)
C25	0.0233 (8)	0.0324 (10)	0.0243 (8)	−0.0026 (7)	0.0044 (6)	−0.0078 (7)
C26	0.0212 (7)	0.0263 (9)	0.0241 (9)	−0.0010 (6)	0.0048 (6)	0.0003 (7)
C27	0.0223 (7)	0.0296 (9)	0.0248 (9)	−0.0024 (7)	0.0049 (6)	0.0038 (7)
C28	0.0447 (10)	0.0295 (10)	0.0590 (12)	−0.0009 (8)	0.0129 (9)	−0.0138 (9)

### Geometric parameters (Å, °)

**Table:**

O1—C1	1.2272 (17)	C16—H16A	0.950
C1—C6	1.4954 (19)	C17—H17A	0.980
C1—C2	1.5022 (19)	C17—H17B	0.980
C2—C10	1.3378 (19)	C17—H17C	0.980
C2—C3	1.5062 (19)	C18—H18A	0.980
C3—C4	1.5198 (19)	C18—H18B	0.980
C3—H3A	0.990	C18—H18C	0.980
C3—H3B	0.990	C20—C21	1.4665 (19)
C4—C5	1.5140 (19)	C20—H20A	0.950
C4—H4A	0.990	C21—C26	1.3970 (19)
C4—H4B	0.990	C21—C22	1.4067 (19)
C5—C6	1.5049 (19)	C22—C23	1.381 (2)
C5—H5A	0.990	C22—C27	1.4981 (19)
C5—H5B	0.990	C23—C24	1.392 (2)
C6—C20	1.3402 (18)	C23—H23A	0.950
C10—C11	1.4683 (19)	C24—C25	1.386 (2)
C10—H10A	0.950	C24—C28	1.503 (2)
C11—C16	1.3983 (19)	C25—C26	1.381 (2)
C11—C12	1.4033 (19)	C25—H25A	0.950
C12—C13	1.3824 (19)	C26—H26A	0.950
C12—C17	1.4973 (19)	C27—H27A	0.980
C13—C14	1.3858 (19)	C27—H27B	0.980
C13—H13A	0.950	C27—H27C	0.980
C14—C15	1.3878 (19)	C28—H28A	0.980
C14—C18	1.499 (2)	C28—H28B	0.980
C15—C16	1.3829 (19)	C28—H28C	0.980
C15—H15A	0.950		
			
O1—C1—C6	120.80 (13)	C11—C16—H16A	119.3
O1—C1—C2	120.39 (13)	C12—C17—H17A	109.5
C6—C1—C2	118.80 (12)	C12—C17—H17B	109.5
C10—C2—C1	117.17 (12)	H17A—C17—H17B	109.5
C10—C2—C3	124.17 (12)	C12—C17—H17C	109.5
C1—C2—C3	118.58 (12)	H17A—C17—H17C	109.5
C2—C3—C4	111.52 (11)	H17B—C17—H17C	109.5
C2—C3—H3A	109.3	C14—C18—H18A	109.5
C4—C3—H3A	109.3	C14—C18—H18B	109.5
C2—C3—H3B	109.3	H18A—C18—H18B	109.5
C4—C3—H3B	109.3	C14—C18—H18C	109.5
H3A—C3—H3B	108.0	H18A—C18—H18C	109.5
C5—C4—C3	109.10 (12)	H18B—C18—H18C	109.5
C5—C4—H4A	109.9	C6—C20—C21	128.42 (13)
C3—C4—H4A	109.9	C6—C20—H20A	115.8
C5—C4—H4B	109.9	C21—C20—H20A	115.8
C3—C4—H4B	109.9	C26—C21—C22	118.20 (13)
H4A—C4—H4B	108.3	C26—C21—C20	121.40 (12)
C6—C5—C4	111.84 (11)	C22—C21—C20	120.25 (12)
C6—C5—H5A	109.2	C23—C22—C21	118.92 (13)
C4—C5—H5A	109.2	C23—C22—C27	119.96 (13)
C6—C5—H5B	109.2	C21—C22—C27	121.12 (12)
C4—C5—H5B	109.2	C22—C23—C24	122.82 (13)
H5A—C5—H5B	107.9	C22—C23—H23A	118.6
C20—C6—C1	117.23 (12)	C24—C23—H23A	118.6
C20—C6—C5	124.42 (12)	C25—C24—C23	117.97 (13)
C1—C6—C5	118.35 (11)	C25—C24—C28	121.41 (14)
C2—C10—C11	128.14 (13)	C23—C24—C28	120.61 (14)
C2—C10—H10A	115.9	C26—C25—C24	120.23 (14)
C11—C10—H10A	115.9	C26—C25—H25A	119.9
C16—C11—C12	118.44 (12)	C24—C25—H25A	119.9
C16—C11—C10	121.86 (13)	C25—C26—C21	121.85 (13)
C12—C11—C10	119.65 (12)	C25—C26—H26A	119.1
C13—C12—C11	118.72 (12)	C21—C26—H26A	119.1
C13—C12—C17	119.94 (13)	C22—C27—H27A	109.5
C11—C12—C17	121.33 (12)	C22—C27—H27B	109.5
C12—C13—C14	123.18 (13)	H27A—C27—H27B	109.5
C12—C13—H13A	118.4	C22—C27—H27C	109.5
C14—C13—H13A	118.4	H27A—C27—H27C	109.5
C13—C14—C15	117.73 (13)	H27B—C27—H27C	109.5
C13—C14—C18	120.01 (13)	C24—C28—H28A	109.5
C15—C14—C18	122.24 (13)	C24—C28—H28B	109.5
C16—C15—C14	120.44 (13)	H28A—C28—H28B	109.5
C16—C15—H15A	119.8	C24—C28—H28C	109.5
C14—C15—H15A	119.8	H28A—C28—H28C	109.5
C15—C16—C11	121.47 (13)	H28B—C28—H28C	109.5
C15—C16—H16A	119.3		
			
O1—C1—C2—C10	−0.3 (2)	C12—C13—C14—C15	0.74 (19)
C6—C1—C2—C10	178.86 (13)	C12—C13—C14—C18	−177.85 (12)
O1—C1—C2—C3	176.57 (15)	C13—C14—C15—C16	−1.32 (19)
C6—C1—C2—C3	−4.3 (2)	C18—C14—C15—C16	177.24 (13)
C10—C2—C3—C4	148.79 (14)	C14—C15—C16—C11	0.5 (2)
C1—C2—C3—C4	−27.81 (19)	C12—C11—C16—C15	0.91 (19)
C2—C3—C4—C5	60.25 (16)	C10—C11—C16—C15	178.63 (12)
C3—C4—C5—C6	−60.88 (15)	C1—C6—C20—C21	174.29 (13)
O1—C1—C6—C20	2.1 (2)	C5—C6—C20—C21	−6.7 (2)
C2—C1—C6—C20	−177.03 (13)	C6—C20—C21—C26	−39.0 (2)
O1—C1—C6—C5	−176.96 (15)	C6—C20—C21—C22	145.61 (14)
C2—C1—C6—C5	3.9 (2)	C26—C21—C22—C23	−0.09 (18)
C4—C5—C6—C20	−150.24 (14)	C20—C21—C22—C23	175.44 (11)
C4—C5—C6—C1	28.73 (18)	C26—C21—C22—C27	178.95 (12)
C1—C2—C10—C11	−179.33 (13)	C20—C21—C22—C27	−5.52 (19)
C3—C2—C10—C11	4.0 (2)	C21—C22—C23—C24	−0.58 (19)
C2—C10—C11—C16	43.5 (2)	C27—C22—C23—C24	−179.63 (12)
C2—C10—C11—C12	−138.83 (15)	C22—C23—C24—C25	0.6 (2)
C16—C11—C12—C13	−1.47 (18)	C22—C23—C24—C28	−179.46 (13)
C10—C11—C12—C13	−179.23 (12)	C23—C24—C25—C26	0.0 (2)
C16—C11—C12—C17	179.60 (12)	C28—C24—C25—C26	−179.90 (13)
C10—C11—C12—C17	1.84 (19)	C24—C25—C26—C21	−0.7 (2)
C11—C12—C13—C14	0.66 (19)	C22—C21—C26—C25	0.71 (19)
C17—C12—C13—C14	179.61 (12)	C20—C21—C26—C25	−174.76 (12)

### Hydrogen-bond geometry (Å, °)

**Table:**

Cg1 and Cg2 are the centroids of the C21–C26 and C11–C16 rings, respectively.

**Table:**

*D*—H···*A*	*D*—H	H···*A*	*D*···*A*	*D*—H···*A*
C17—H17B···Cg1^i^	0.98	3.00	3.532 (1)	154
C17—H17C···Cg1^ii^	0.98	2.62	3.469 (1)	111
C27—H27B···Cg2^iii^	0.98	2.64	3.486 (1)	145
C27—H27C···Cg2^iv^	0.98	2.80	3.510 (1)	130

Symmetry codes: (i) −*x*, *y*+1/2, −*z*+1/2; (ii) −*x*+1, *y*+1/2, −*z*+1/2; (iii) −*x*, *y*−1/2, −*z*+1/2; (iv) −*x*+1, *y*−1/2, −*z*+1/2.
